# Early experience and perioperative risk of GammaTile for upfront brain metastases: Report from a prospective multicenter study

**DOI:** 10.1093/noajnl/vdae156

**Published:** 2024-09-10

**Authors:** Sabrina L Zeller, Sauson Soldozy, Shaye Busse, Clark C Chen, Andrew Venteicher, Clara Ferreira, Kathryn Dusenbery, Stuart Lee, Matthew Sean Peach, Vincent DiNapoli, Rupesh Kotecha, Manmeet S Ahluwalia, Kimberly Bojanowski-Hoang, Simon J Hanft

**Affiliations:** Department of Neurosurgery, Westchester Medical Center, Valhalla, New York, USA; Department of Neurosurgery, Westchester Medical Center, Valhalla, New York, USA; Department of Neurosurgery, Westchester Medical Center, Valhalla, New York, USA; Department of Neurosurgery, Warren Alpert School of Medicine, Rhode Island Hospital, Brown University, Providence, Rhode Island, USA; Department of Neurosurgery, University of Minnesota, Minneapolis, Minnesota, USA; Department of Radiation Oncology, University of Minnesota, Minneapolis, Minnesota, USA; Department of Radiation Oncology, University of Minnesota, Minneapolis, Minnesota, USA; Department of Neurosurgery, ECU Health, Greenville, North Carolina, USA; Department of Radiation Oncology, ECU Health, Greenville, North Carolina, USA; Department of Neurosurgery, Mayfield Brain & Spine, Cincinnati, Ohio, USA; Department of Radiation Oncology, Miami Cancer Institute, Baptist Health South Florida, Miami, Florida, USA; Department of Radiation Oncology, Miami Cancer Institute, Baptist Health South Florida, Miami, Florida, USA; Department of Neurosurgery, Emory University, Atlanta, Georgia, USA; Department of Neurosurgery, Westchester Medical Center, Valhalla, New York, USA

**Keywords:** brain metastases, GammaTile, brachytherapy, Cesium-131

## Abstract

**Background:**

GammaTile (GT), a form of brachytherapy utilizing cesium-131 seeds in a bioresorbable collagen tile, has gained popularity for the treatment of recurrent intracranial tumors and more recently for newly diagnosed metastases. This study reports early experience utilizing GT in upfront brain metastases with a focus on clinical applications and perioperative safety.

**Methods:**

The STaRT Registry (NCT04427384) was queried for all patients receiving GT for upfront metastases from August 2021 to August 2023. Data regarding patient demographics, procedure details, and adverse events (AEs) were extracted and analyzed.

**Results:**

Twenty-eight patients, median age 65 years (range 28–81), with 30 treated metastases were reported from 6 institutions. Patients had 2.8 metastases on average (range 1–15) at the time of surgery; however, most patients had a single metastasis (60.7%). The mean diameter of treated metastases was 3.4 cm (range 1.5–4.7). A median of 4.0 tiles (range 1–10) were used per tumor. The median follow-up was 3.0 months (range 1.0–11.2) with 6 attributed AEs (21.4%), including 1 grade ≥ 3 (infection). In the immediate postoperative period (<14 days), 2 patients reported pain or headache, and 1 reported facial edema. One patient developed seizures on postoperative day 8 requiring medication. At 1-month follow-up, there was 1 superficial wound infection, in a previously colonized patient, requiring surgical intervention without explantation of tiles. At 3-month follow-up, 1 patient reported facial pain not requiring treatment. There were no symptomatic hematomas.

**Conclusions:**

GT demonstrates a favorable safety profile in upfront brain metastases with a 3.6% rate of serious AEs (grade ≥ 3) within 90 days of the procedure.

## Lay Summary

GammaTile is a new treatment for brain tumors that involves implanting a small device filled with radioactive material during surgery. This device targets and kills cancer cells directly in the brain. The authors of this study wanted to see whether GammaTile would be safe to use for patients who have had cancer spread to the brain from elsewhere in the body. To do this, they combined data from 28 patients who had GammaTile implanted after their metastases were surgically removed. On average, each patient had 3 separate metastases in the brain, and the treated tumors were about 3.4 cm in size. Their results showed that 21% of patients had complications from GammaTile; however, only 3.6% were considered serious by the authors. Complications included pain, headaches, facial swelling, seizures, and a superficial infection that required additional surgery. Overall, the authors believe that GammaTile seems to be a safe option for treating newly diagnosed brain metastases.

**Key Points**

- GammaTile shows a favorable perioperative safety profile in upfront brain metastases.
- No adverse events in this study were directly attributable to GammaTile.

**Importance of the Study**

While GammaTile has recently gained popularity in recurrent intracranial tumors, this is the first study to report an early multicenter experience of utilizing GammaTile in newly diagnosed brain metastases. The overall early safety profile in a 28-patient cohort is favorable, revealing a 3.6% rate of serious AEs and 21.4% of any AE. None of the AEs in the cohort were definitively related to GammaTile placement and had only a possible correlation.

Brain metastases are the most common malignant brain tumors in adults, affecting up to 30% of cancer patients.^1^ The incidence of patients with brain metastases varies based on the primary diagnosis, with an incidence exceeding 10% for patients diagnosed with melanoma (28.2%), various lung malignancies (15.5%–26.8%), and renal cell carcinoma (10.8%).^2^ The advent of improved imaging diagnostics along with prolonged life expectancy secondary to advances in cancer therapies have contributed to a rise in the incidence of brain metastases. While management is often dependent on the primary malignancy, surgical intervention is indicated when the metastasis is symptomatic or producing a significant mass effect, or when tissue diagnosis is needed.^3^ Adjuvant radiation therapy for brain metastases reduces local recurrence of surgically resected tumors.^4,5^ Historically, whole-brain radiation therapy was typically employed; however, given its neurotoxic effects, it has largely fallen out of favor for more precise stereotactic radiosurgery (SRS).^6^

GammaTile (GT; GT Medical Technologies Inc) is an FDA-cleared brachytherapy device utilizing cesium-131 seeds embedded in a collagen carrier. For GT specifically, there is improved homogeneous radiation dose delivery thought to be due to the 3-mm collagen border between the seed and target tissue.^7,8^ The collagen tiles also maintain seed spacing, decreasing seed migration and lowering the risk of underdosing some areas, which could lead to tumor recurrence.^9^ There is also a lower risk of overdosing in some areas from seed clumping as is possible in other forms of brachytherapy, reducing the risk of radiation necrosis.^10^

GT has gained popularity for the treatment of recurrent intracranial metastases and recurrent primary brain tumors. A subset of brain metastases shows highly aggressive behavior despite surgical resection, thus resulting in recurrence while awaiting radiosurgery.^1^ One study explored the use of GT brachytherapy in this subset of patients, as defined by symptomatic recurrence prior to radiosurgery or tumor growth by 25% on preoperative imaging, and demonstrated favorable results in a cohort of 10 patients who showed no local recurrence or complications after implantation.^1^ Furthermore, a recent study evaluating the use of GT in recurrent glioblastoma (GBM) found 6-, 12-, and 18-month survival rates of 75%, 46%, and 29%, respectively, compared to a 12-month post-treatment survival rate of 44% for other forms of brachytherapy (I-125 and Ir-192) and 34% for external beam radiation therapy according to a recent meta-analysis of over 60 000 patients.^11,12^

The STaRT Registry (NCT04427384) is a multicenter multi-histology prospective cohort of patients who have undergone intracranial tumor resection with placement of GTs. Here we report the first cohort of patients for whom GT was used for upfront brain metastases from the Registry. We focus on the clinical applications and perioperative safety profile in this population.

## Materials and Methods

### Patient Selection and Data Collection

The STaRT Registry (NCT04427384), a multicenter IRB-approved compendium of prospectively enrolled patients who have undergone intracranial tumor resection followed by GT placement, was assessed for all patients with upfront metastasis from August 2021 to August 2023. Deidentified data regarding patient demographics, procedure details, and adverse events (AEs) were extracted and analyzed. Grading of AEs was determined in accordance with CTCAE Version 5.^13^ Data were stored in a password-protected spreadsheet without identifiable patient information.

### GammaTile Specifications and Technique

Following surgical resection, the tumor cavity is lined with a single layer of GTs. Each GT consists of a 20 × 20 × 4 mm collagen square embedded with 4 titanium-encapsulated Cs-131 seeds, which each provide a fixed source strength of 3.5 cGy cm^2^ h^−1^ on the day of implant and have a half-life of 9.7 days. The bioresorbable collagen carrier is permanently implanted and provides fixed spacing of the Cs-131 seeds 10 mm from each other and 3 mm from the cavity surface. This GT design allows a dose of approximately 60 Gy to a depth of 5 mm from the surface, though this can vary based on the cavity and number of tiles used.^8^

The biologically effective dose (BED), based on the linear-quadratic model, corresponding to 60 Gy from a permanently implanted Cs-131 GT implant (BED of 51.5 Gy) is similar to that of the commonly used hypofractionated stereotactic radiation therapy schedule of 30 Gy delivered in 5 fractions (BED of 48.0 Gy) for early responding tissues such as brain metastases (α/β = 10).^14,15^ A recent dosimetric comparison between GT and external beam stereotactic radiotherapy approaches (including CyberKnife, GammaKnife, and proton therapy) demonstrates equivalent or greater BED up to at least 3 mm from the resection cavity edge from GT compared to the radiosurgery approaches.^16^

### Statistical Analysis

Analysis is presented on a per-patient basis; results are presented as mean ± standard deviation, median, and range.

## Results

### Patient Demographics

Twenty-eight patients were included in the study from 6 institutions with a median age of 65 years (range 28–81), as shown in Table 1. There were a total of 30 treated metastases, including 2 patients treated for 2 metastases concurrently. There was a slight male predominance of 53.6%.

**Table 1.** Summary of Demographic Information for Patients Treated With GammaTile for Newly Diagnosed Brain Metastases

Patient Demographics/Characteristics	
Patients (*n*)	28
Tumors implanted (*n*)	30
Age (median)	65 (range 28–81)
Sex (*n*, %)	
Male	15 (53.6%)
Female	13 (46.4%)
Race (*n*, %)	
White	24 (85.7%)
Black	4 (14.3%)
BMI (median)	26.5 (range 17.2–50.6)
Tumor characteristics	
Primary malignancy (*n*, %)	3.4 (range 1.5–5.2)
Lung	12 (42.9%)
Melanoma	4 (14.3%)
Breast	3 (10.7%)
Renal	3 (10.7%)
Colon	1 (3.6%)
Other	5 (17.8%)
Total number of mets (median)	1 (range 1–15)
Number of mets treated (*n*, %)	
1	26 (92.9%)
2	2 (7.1%)
Maximum diameter (cm, median)	3.4 (range 1.5–5.2)
Tiles used (median)	4.0 (range 1.0–10.0)

Abbreviations: BMI = body mass index; mets = metastases.

Only a single patient had received prior cranial radiation, a patient with primary lung cancer who underwent 3000 cGy prophylactic cranial irradiation in 15 fractions 3 years prior to GT placement.

### Tumor Characteristics and Operative Details

The most common primary malignancies were lung (42.9%), melanoma (14.3%), breast (10.7%), and renal (10.7%). Other less-represented primaries included colon and esophageal. Patients had an average of 2.8 ± 3.4 identified metastases (median: 1, range: 1–15 metastases) at the time of surgery; however, GT was most commonly utilized in patients who had a single metastasis (60.7%) at the time of treatment.

**Figure 1.**
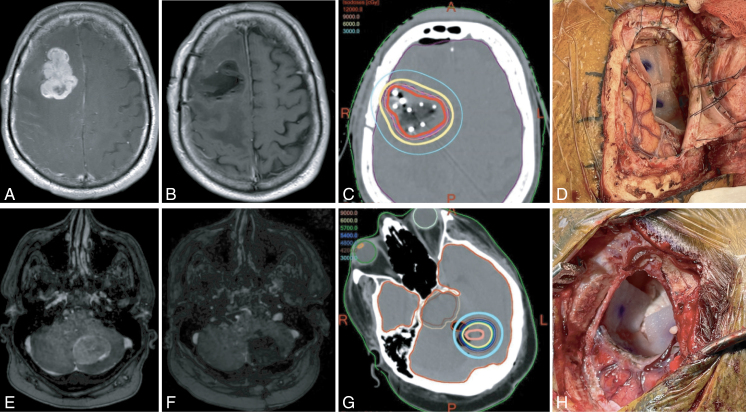
Case examples of 2 patients who underwent surgical resection and GammaTile placement for newly diagnosed brain metastases. A right frontal melanoma metastasis is demonstrated by the preoperative axial T1-weighted post-contrast MRI (A), immediate postoperative axial T1-weighted post-contrast MRI (B), GammaTile radiation therapy dosimetry (C), and intraoperative photograph of resection cavity lined with GammaTile (D). A left cerebellar non–small cell lung cancer metastasis is demonstrated by the same images, respectively (E, F, G, H).

The average maximum diameter of treated metastases was 3.4 ± 1.1 cm (median: 3.4 cm, range 1.5–4.7 cm). Tumors were most commonly located in the frontal (*n* = 14, 46.7%), parietal (*n* = 8, 26.7%), and temporal lobes (*n* = 4, 13.3%); there were 2 tumors in the occipital lobe (6.7%) and cerebellum (6.7%). Gross total resection was achieved for 96.7%. On average, 4.7 ± 2.3 tiles (median: 4.0 tiles, range: 1–10 tiles) were used per tumor. Two illustrative cases are provided in Figure 1.

### Follow-Up and Complications

The cohort had a median follow-up of 3.0 months (range 1.0–11.2) with 6 reported AEs (21.4% of patients), with all characterized as possibly attributable to GT, and including only 1 grade ≥ 3 using CTCAE Version 5 criterion (Table 2).^13^ No AEs in the registry were categorized as definitely attributable to GT placement. In the immediate postoperative period, 2 patients reported pain or headache, and 1 reported facial edema; each of these complications was attributable to standard postoperative craniotomy symptoms and were self-resolving. Patients were not routinely administered prophylactic anticonvulsants following GT placement; however, those who had previously experienced seizures were continued on their medications. One patient developed seizures on postoperative day 8 requiring medication but no further intervention; seizures were likely related to intracranial disease with multiple remaining metastases rather than implantation of the tiles.

**Table 2.** Adverse Events Reported in Patients Who Received GammaTile for Upfront Brain Metastases

Pt	Time After Surgery (days)	AE CTCAE Grade	Type	Action Taken	Related to Surgery? (Definite, Probable, Possible)	Related to GT? (Definite, Probable, Possible)
1	8	2	Seizure	Medication	Possible	Possible
2	26	3	MRSA wound infection	Surgical intervention	Definite	Possible
3	0	2	Headache	Medication	Definite	Possible
4	3	1	Facial edema	None	Definite	Possible
5	127	1	Facial pain	Nonmedical treatment	Definite	Possible
6	2	2	Postoperative pain	Medication	Definite	Possible

Abbreviations: AE = adverse event; CTCAE = Common Terminology Criteria for Adverse Events; GT = GammaTile; MRSA = methicillin-resistant *Staphylococcus aureus*; Pt = patient.

At 1-month follow-up, there was 1 superficial wound infection requiring surgical intervention. The patient was known to be colonized with methicillin-resistant *Staphylococcus aureus* (MRSA) and had a history of developing superficial MRSA infections with all prior surgeries. The infection was contained to the subcutaneous space requiring superficial washout of the incision without exploration of the surgical cavity or explantation of tiles. By the 6-month follow-up, the infection had fully resolved, and the patient remained on suppressive antibiotics indefinitely per the patient’s infectious disease physician.

At 3-month follow-up, 1 patient reported facial pain of unknown etiology not requiring treatment. The pain was specifically described as jaw soreness and was possibly related to surgical resection, though was unlikely to be related to GT placement.

There were no symptomatic hematomas or adverse radiation effects identified in this cohort. The 30-day mortality rate was 3.6% (*n* = 1); a septuagenarian experienced cardiopulmonary failure secondary to medical causes distinct from the surgery and index hospitalization.

## Discussion

This study is the first to report the perioperative safety of utilizing GT in newly diagnosed brain metastases. Several factors make brachytherapy a favorable mode of treatment for intracranial neoplasms after surgical resection. As the contemporary management of upfront brain metastases shows a trend toward earlier radiation, with preoperative or early postoperative SRS, intraoperative initiation of radiation represents a promising alternative.^17–19^ Surgical removal and initiation of radiation treatment with brachytherapy allows treatment when the tumor burden is at a minimum, as opposed to a delay of up to 3–4 weeks to allow for surgical recovery and wound healing prior to postoperative radiotherapy.^20^ While postoperative SRS often requires expansion of the cavity margins due to the uncertainty of postoperative target borders, the use of a radioactive implant placed immediately after tumor resection enables precise targeting of radiation.^18,19,21^ With a permanently implanted mode of radiotherapy, patients are not required to return for multiple outpatient radiation treatments, which reduces hurdles such as transportation, repeat imaging, insurance authorizations for both repeat MRI and the planned radiotherapy, along other care-related issues.^22^ This also ensures 100% compliance with therapy.^23^

GT demonstrates a favorable safety profile in upfront brain metastases with an overall AE rate of 21.4% and a 3.6% rate of serious AEs (grade ≥ 3). While the reported AEs were possibly related to GT, they may also be attributable to the nature of the disease or surgery itself. Several patients reported pain around the incision site, headaches, or facial swelling, which represent self-limited symptoms expected during the postoperative course. A single patient (3.6%) developed seizures on postoperative day 8; however, since the patient was harboring multiple brain metastases, the seizure activity is more likely attributable to the patient’s intracranial disease burden rather than GT placement. No other patient in our series experienced seizures; although, more data are necessary to clarify seizure risk secondary to GT placement in patients with brain metastases. Current literature suggests seizure risk to be low, which is in line with the findings of this study. In a recent systematic review by Xiang et al. including 26 studies assessing brachytherapy in recurrent GBM, only 6 studies reported seizures as AEs with rates ranging from 0.7% to 13.3%.^24^

In this cohort, 1 patient previously colonized with MRSA suffered a superficial infection, requiring wound washout and subsequent antibiotic treatment. Of note, the tiles were not removed during the wound washout. The infection had resolved by the 6-month follow-up without further complications. The resolution of this infection without the removal of GT suggests that such removal is not essential if the infection is restricted to the superficial compartment. However, in another case series, Dharnipragada et al. reported on a patient with a known *S. aureus* wound infection after an initial brain metastasis resection without GT placement; the patient completed a 6-week course of antibiotics.^1^ Surveillance MRI revealed significant tumor growth during this 6-week period, and the patient underwent a second resection with GT placement, which unfortunately was complicated by a second *S. aureus* infection. Subsequently, the patient underwent a second wound washout and GT removal. The wound infection resolved following an extended course of antibiotic treatment. As these 2 cases constitute the entirety of the current literature, the optimal management strategy for infection post-GT placement regarding the necessity of GT removal remains unclear and awaits future investigations.

In general, the overall complication rate of craniotomy for tumor resection has been reported as 7%.^25^ With the inclusion of postoperative nausea and vomiting, this rate can approach 31%.^26^ The complication rate with GT is comparable, suggesting the safety of GT in conjunction with standard craniotomy for resection. Furthermore, adjuvant radiation therapy is the standard of care for resected brain metastases, compounding the additional risks of postoperative radiotherapy to the risk of craniotomy alone, including potential delays to treatment due to the patient’s clinical course and increased treatment volumes or radiation necrosis secondary to unclear tumor margins.^18,19^ Of note, similar to GT, the advent of preoperative SRS has also addressed some of these risks associated with postoperative SRS; however, there are logistical hurdles to preoperative SRS (patients who are acutely symptomatic, uncertainty as to the timing of surgery following SRS).^18^

While this study reports the novel use of GT in newly diagnosed brain metastases, GT has already been growing in popularity in recurrent brain metastases and glioblastoma. The safety of GT for upfront metastases when compared to GT for recurrent metastases is thought to be comparable. An analysis of outcomes among patients treated with GT as part of a larger prospective study found no wound complications, and only 2 patients developed radiation changes, both among patients who had previously received SRS to the same site, and neither requiring long-term steroid use.^27^ Furthermore, brachytherapy has been utilized in upfront metastases as well. A matched-pair analysis of clinical outcomes after cesium-131 brachytherapy in stranded form versus SRS for upfront brain metastases found 3.3% (1/30) of brachytherapy patients experienced radiation necrosis, compared to 10.0% (6/60) among SRS patients.^28^ Notably, while it is relatively early in the follow-up period, there was no radiation necrosis/ARE in this cohort, which is consistent with the Dharnipragada et al. cohort of radiation-naive patients with brain metastases treated with resection and GT.^1^ This patient cohort requires continued follow-up to evaluate radiation necrosis/ARE.

These findings are the result of a multicenter study; however, the scope of this paper is to report on the early experience of GT in this novel population. Therefore, results are limited due to the small sample size and short-term follow-up. Data collection is ongoing, and further follow-up will be required to evaluate outcomes and long-term complications, such as local control and rates of radiation necrosis. Efficacy data will be presented as part of a planned interim analysis. Furthermore, there is a currently enrolling phase 3 randomized study comparing GT to postoperative stereotactic radiotherapy for resected brain metastases (NCT04365374). In the meantime, we believe reporting the perioperative safety data on this prospective registry is helpful to the field.

In conclusion, this study presents the profile of patients receiving GT for newly diagnosed brain metastases. Early experience in this novel population demonstrates a favorable safety profile to date. Further research will be required to determine efficacy and outcomes.
